# Annealing Effects in Twin-Roll Cast AA8006 Aluminium Sheets Processed by Accumulative Roll-Bonding

**DOI:** 10.3390/ma7128058

**Published:** 2014-12-15

**Authors:** Miroslav Cieslar, Michaela Poková

**Affiliations:** Faculty of Mathematics and Physics, Charles University in Prague, Ke Karlovu 5, CZ-121 16 Prague 2, Czech Republic; E-Mail: pokova@karlov.mff.cuni.cz

**Keywords:** accumulative roll-bonding, AA8006, recrystallization, twin-roll cast

## Abstract

Ultrafine grained sheets were prepared from a twin-roll cast AA8006 aluminium alloy using accumulative roll-bonding process at room temperature. The evolution of microstructure of sheets after three accumulative roll-bonding passes during isochronal annealing with a constant step of 20 °C/20 min was studied by light and electron microscopy. The influence of the resulting microstructure on mechanical properties was monitored by microhardness measurements. The microhardness increases when the material is annealed up to 160 °C. Above this temperature a fast drop of microhardness occurs followed by a negligible variation at annealing temperatures exceeding 300 °C. In order to map continuously the microstructure changes during annealing, the *in situ* TEM experiments in the heating stage were performed as a supplement to *post-mortem* TEM observations.

## 1. Introduction

Mechanical properties of metallic materials are very sensitive to the grain size and its reduction increases the yield stress and the tensile strength according to the Hall-Petch relationship [1,2]. In the last decade, the bulk nano and ultrafine grained materials have received considerable scientific and application attention. Nowadays, severe plastic deformation (SPD) is most frequently used for the grain refinement [3,4]. The basic principle of the SPD process consists in inducing an extremely high plastic strain into the material resulting in a substantial grain refinement and improved strengths. The main advantage of the majority of the SPD methods is that no shape and dimension changes occur during the straining, which plays an important role when large volumes of bulk ultrafine grained (UFG) materials have to be produced.

Accumulative roll-bonding (ARB) belongs to one of the most popular SPD techniques, which does not require any special equipment [5,6]. Since ARB enables the production of large amounts of UFG sheets of various compositions (including composite materials) [7,8], which might be further thermo-mechanically treated, its implementation in the industrial practice is more probable than that of other SPD techniques such as equal-channel angular pressing or high-pressure torsion [9,10]. The grain structure usually formed during the ARB processing is lamellar [11]. Its homogeneity and final thermal stability in aluminium alloys depends on the processing temperature, number of ARB cycles but also on the grain size of the initial material and the size and distribution of coarse primary particles which are generally present in the ingot cast and cold-rolled sheets. Therefore, the thermal stability and homogeneity is improved in materials with finer particles and small grain size which is typical for continuously twin-roll cast (TRC) aluminium alloys [12,13,14,15,16]. Because the use of TRC aluminium materials in the industry is continuously more and more frequent the feasibility conditions of ARB processing and thermo-mechanical post-processing become an important part of the SPD materials investigation.

AA8006 foils are often used as fins in car radiators. Nevertheless, increasing demands of the industry on formability, mechanical properties and corrosion resistance has evoked an intensive investigation of sheets and foils with ultrafine grained structure. Although TRC aluminium sheets are optimal candidates for further grain refinement, generally only a limited number of data is available on SPD processed TRC materials. Recently first results on microstructure and mechanical properties of ARB sheets prepared from the TRC AA8006 alloy were published [17] and the properties of such materials were compared with conventionally cold-rolled specimens [18]. The main goal of the present study is to map microstructural changes occurring during annealing of the deformed material using the *in situ* Transmission Electron Microscopy (TEM) in the heating stage and to assess the impact of such changes on the room temperature microhardness.

## 2. Experimental Details

A commercial TRC AA8006 type alloy (0.40 Mn, 0.16 Si, 1.51 Fe in wt%) was used in the study. Supplied 2 mm thick sheets were homogenized for 18 h at 610 °C and then annealed at 450 °C for 30 min in order to obtain fine-grained and fully recrystallized material. In the following text the material was denoted as F0.

ARB process involves the repetition of four steps: (1) degreasing and brushing with a stainless steel brush; (2) stacking of pieces of 300 mm × 50 mm × 2 mm; (3) joining by Al wires; (4) room temperature rolling with 50% reduction without lubricant (the final thickness of the bonded sheet remains 2 mm). These steps present one ARB cycle which was repeated three times (specimen F3) and for the reference microhardness measurements also five times (specimen F5). Etching by the Barker’s anodizing solution and imaging using the polarized light in the light optical microscope (LOM) were used for the grain structure mapping. The distribution of phases was examined by LOM on specimens finally polished by OPS suspension. Dimensions of about 5000 particles were analyzed by the image analyzer in several selected specimen. Specimens for TEM observations were prepared by a twin-jet electro-polishing device in 33% of nitric acid in the methanol at −15 °C. The TEM analyses were carried out at 200 kV with JEOL JEM 2000FX electron microscope equipped with SDD EDS detector. A JEOL standard heating holder was used for the *in situ* annealing experiments. The evolution of mechanical properties was monitored by the microhardness HV_0.1_ measurements. All presented observations were performed in the plane perpendicular to the transversal direction of the sheet. The thermal stability was studied during the step-by-step isochronal annealing ranging from 100 °C to 440 °C with the step 20 °C/20 min. *In situ* TEM observations were performed with the step 50 °C/50 min, which keeps equivalent heating rate as in the rest of experiments. Dislocation densities were estimated by the Ham’s method [19]. The thickness of the foil was measured by a convergent beam method. Standard lineal intercept procedure was used for the subgrain size determination.

## 3. Experimental Results

### 3.1. Mechanical Properties

**Figure 1:**
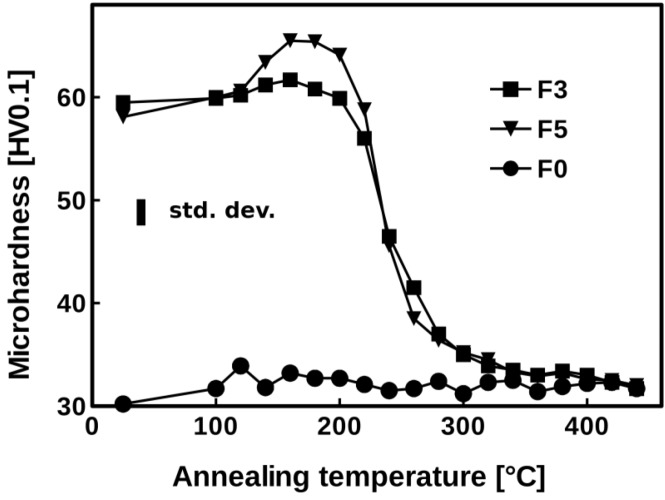
Evolution of microhardness in undeformed (F0) and accumulative roll-bonding (ARB) processed (F3 and F5) specimens during step-by-step annealing.

Vickers microhardness measurements were carried out on the material F0 and ARB processed specimen F3 and reference specimen F5 annealed at different temperatures (Figure 1). In the initial state, the microhardness of both ARB processed specimens F3 and F5 is obviously higher than the one observed in the F0 sheet due to the strain imposed byARB cycles. Microhardness of the F0 specimen, which is fully recrystallized, remains nearly constant within the average experimental error (given by the error bar in Figure 1) during the whole annealing cycle. Unsystematic small variations of this value reflect rather inhomogeneity of mechanical properties along the length of the strip than any changes in the microstructure of the specimen. In the F3 specimen the first variations of microhardness are visible above 120 °C. Microhardness systematically increases and reaches the maximum at 160 °C. This surprising behavior is even more pronounced in the reference specimen F5. Between 160 °C and 300 °C microhardness drops until the value slightly above 30 HV_0.1_ is reached. This value is equal to the F0 final microhardness.

### 3.2. LOM

**Figure 2:**
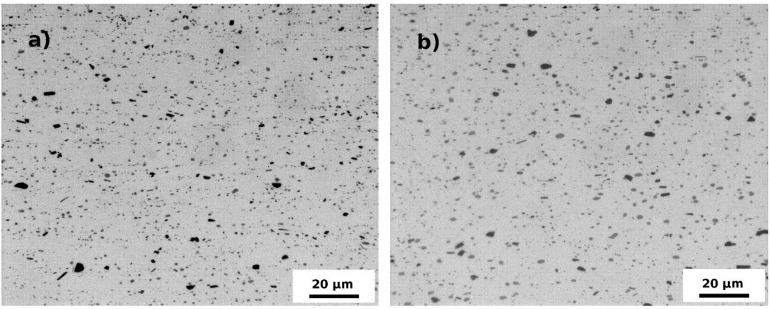
Distribution of dispersoids (**a**) in the F0 specimen in the initial state after homogenization; and (**b**) in the specimen F3 annealed up to 440 °C.

**Figure 3:**
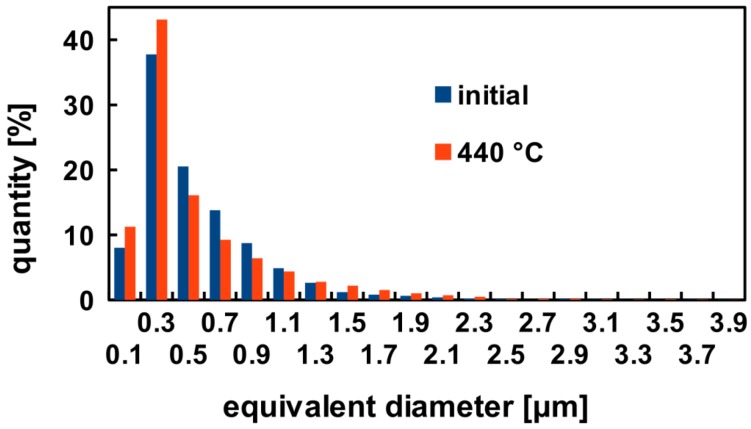
Distribution of particles size in the F0 specimen in the initial state and after step-by-step annealing up to 440 °C.

**Figure 4:**
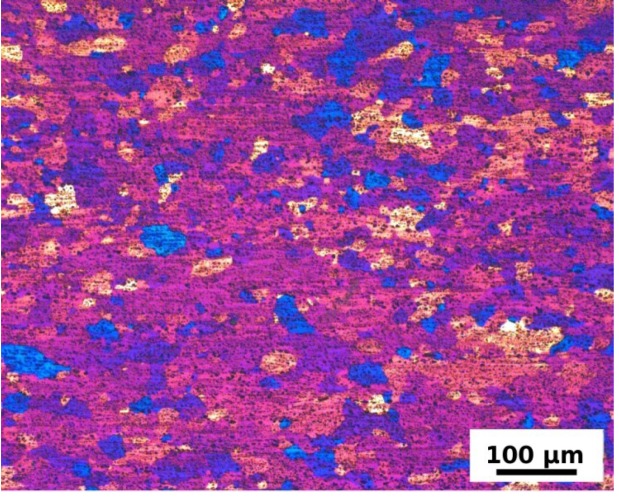
Light optical microscope (LOM) micrograph of the coarse grained structure in the F0 specimen in the initial state.

**Figure 5:**
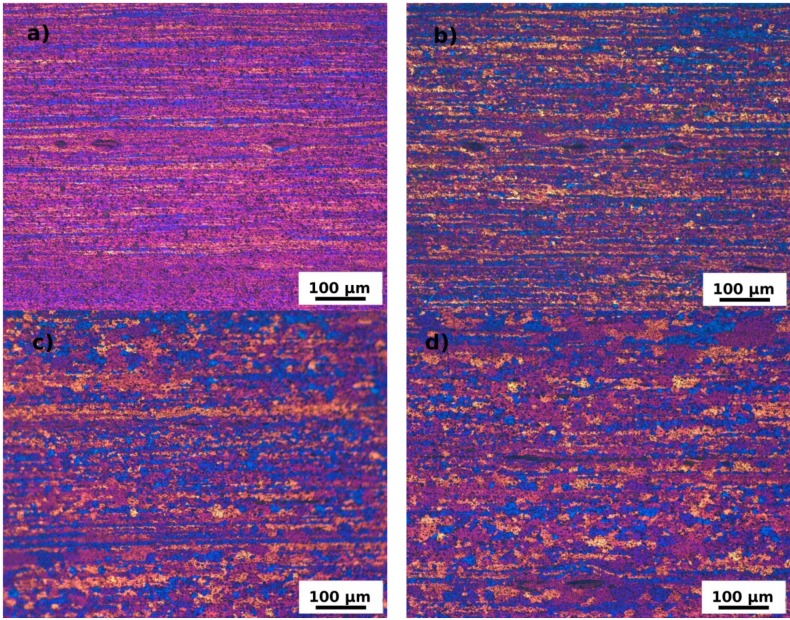
LOM micrographs of the F3 material in the initial state (**a**) after ARB processing; and annealing up to (**b**) 280 °C; (**c**) 380 °C; and (**d**) 440 °C.

Dispersion of coarser (~5 μm) and finer (~0.5–1 μm) particles was observed in all specimens in the initial state (Figure 2a) as well as in the annealed ones (Figure 2b). Their size, volume fraction and distributions were rather unaffected by the ARB process and by the annealing as can be seen in Figure 3. Similarly the fully recrystallized grain structure of the F0 specimen remains untouched by the annealing. No grain coarsening was observed and the size of grains remains constant during the entire annealing cycle (see Figure 4). The ARB processing has obviously on the other hand significant influence on the resulting grain structure. The originally recrystallized equiaxed grains were replaced by flat ones elongated in the rolling direction (Figure 5a). First recrystallized grains were observed at 280 °C (Figure 5b) followed by a partial recrystallization and grain growth at 440 °C (Figure 5c,d). Although the microhardness of the F3 specimen reaches finally the same value as the one observed in the F0 specimen, the initial elongated morphology of deformed grains partially still persists.

### 3.3. TEM

**Figure 6:**
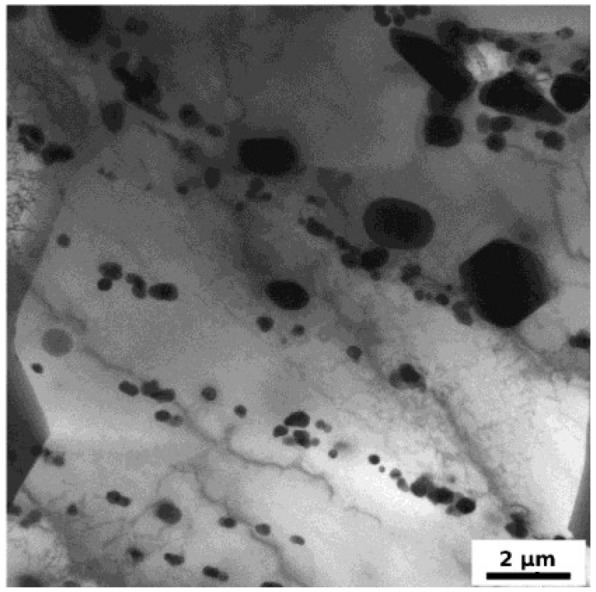
Transmission Electron Microscopy (TEM) micrograph of F0 specimen in the initial state.

**Figure 7:**
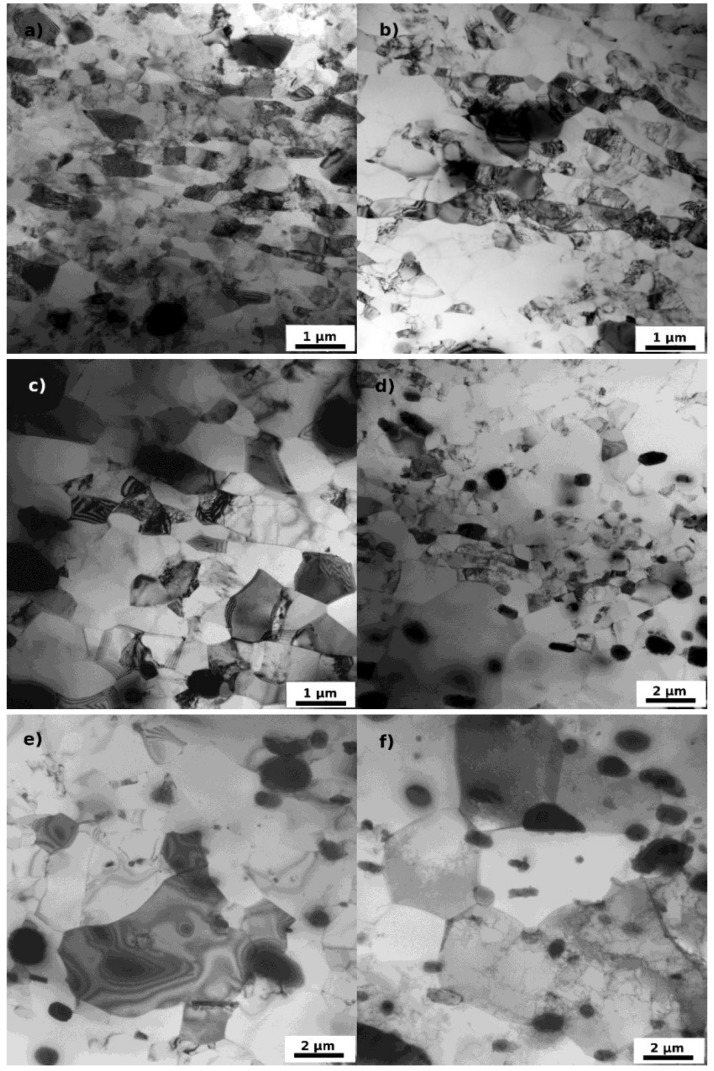
TEM micrographs of F3 material (**a**) after ARB processing; and annealing up to (**b**) 200 °C; (**c**) 240 °C; (**d**) 260 °C; (**e**) 300 °C; and (**f**) 440 °C.

The TEM experiments were performed on specimens in the initial state and on specimens F3 annealed up to several selected temperatures in the same way as specimens for microhardness and LOM experiments. The input material F0 is fully recrystallized with large grains unmeasurable by the TEM. They contain, in accordance with LOM observations, finer and coarser particles (Figure 6) identified from diffraction patterns and by the EDS analysis mainly as cubic α-Al_12_(Fe,Mn)_3_Si phase and less numerous orthorhombic Al_6_(Fe,Mn) phase that are generally observed in AA8006 alloys [12,15]. They were formed and stabilized during the high-temperature pretreatment and therefore any further thermal exposure has no influence on their size or distribution. The ARB processing has on the other hand an important impact on the microstructure. Flat grains elongated in the rolling direction are formed by the process and main features of the deformed microstructure remain unaffected by annealing up to 100 °C. Each original grain is segmented into numerous grains and subgrains (the Kikuchi patterns statistical analysis gives about 50% of high angle grain boundaries) with very low dislocation density in their interiors (about 5 × 10^13^ m/m^3^) and sparsely dispersed dislocation cell walls (Figure 7a).

At higher annealing temperatures the dislocation density decreases below 10^13^ m/m^3^, and at 200 °C nearly all dislocations are annealed out or incorporated in cell-walls and low-angle boundaries (Figure 7b). At 240 °C grains and subgrains are nearly free from cell-walls and further sharpening of existing subgrain boundaries occurs accompanied by the first observable grain growth (Figure 7c). At 260 °C the formation and growth of recrystallized grains can be recognized (Figure 7d) often inhomogeneously along the specimen thickness (note also the different scale). Above this annealing temperature the deformation microstructure is replaced by recrystallized grains (Figure 7e) followed by an imperceptible grain coarsening (Figure 7f). The residual dislocation density observed in Figure 7f appears as an artifact of the TEM specimen preparation. The soft material most probably could not resist the temperature cycling during electrolytical polishing.

**Figure 8:**
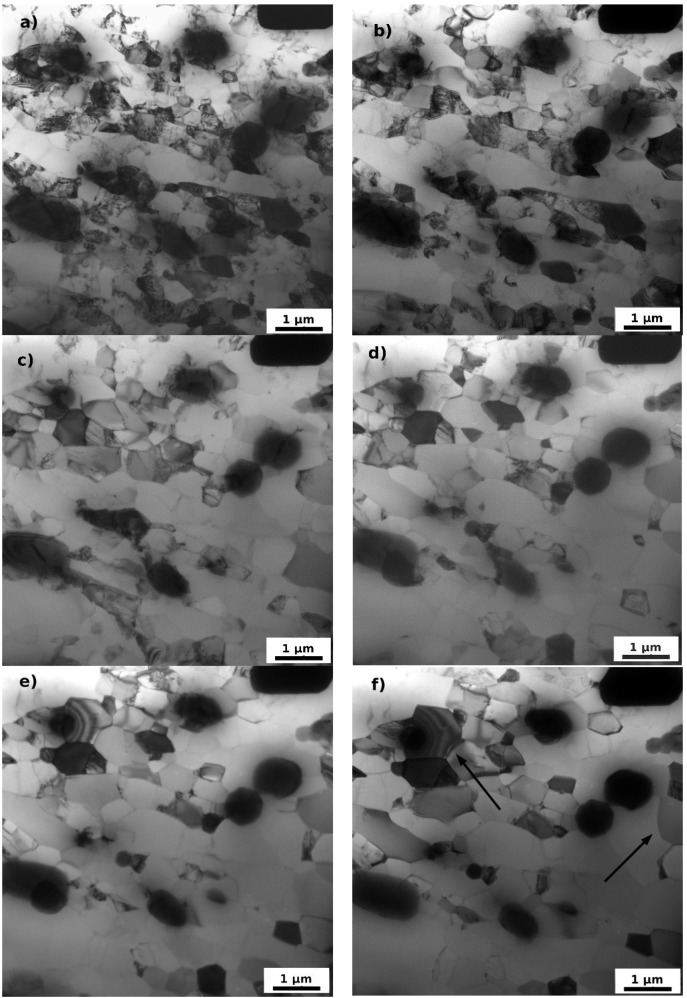
TEM micrographs of F3 material (**a**) after ARB processing; and annealing up to (**b**) 150 °C; (**c**) 200 °C; (**d**) 250 °C; (**e**) 300 °C; and (**f**) 350 °C. Images show the same area in the specimen *in situ* annealed inside the electron microscope.

**Figure 9:**
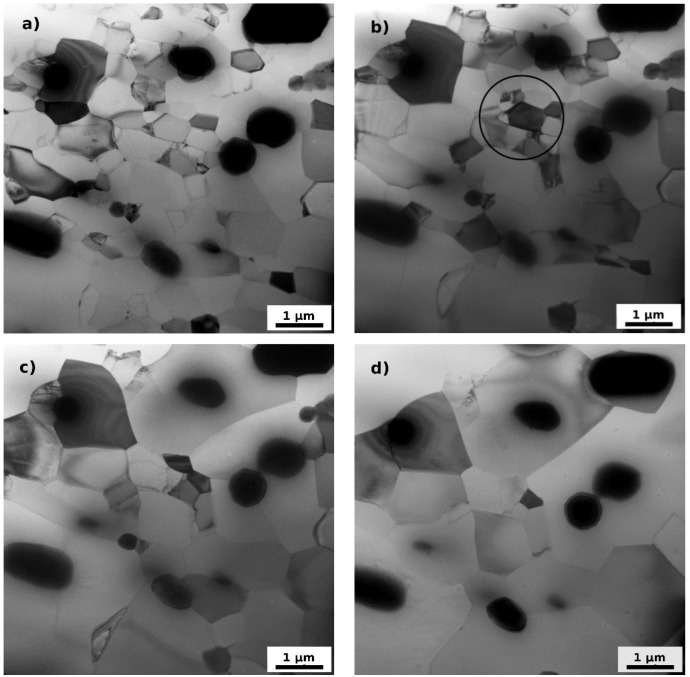
TEM micrographs of F3 material after ARB processing and annealing up to (**a**) 400 °C; (**b**) 450 °C; (**c**) 500 °C; and (**d**) 550 °C. The same area as in Figure 8 is viewed.

In order to study details of processes occurring during annealing the *post-mortem* TEM observations were completed by *in situ* TEM experiments. Figure 8 and Figure 9 summarize received results confirming the main features and temperature intervals of annealing processes observed during post-mortem experiments. All examinations were performed in the same area of the specimen. Figure 8a–d shows clearly the modification of the dislocation substructure with increasing annealing temperature, the gradual embedding of sporadic dislocations into cell-walls and boundaries finalized by the formation of well-defined dislocation-free grains and subgrains at 200 °C (the dislocation density drops to 10^12^ m/m^3^). It is worth mentioning that no subgrain growth occurs in this temperature interval and the prevailing process is the annihilation of dislocations and dissolution of cell-walls. First recognizable growth of recrystallization nuclei occurs at 250 °C (see arrows in Figure 8f). Figure 9 shows the sequential transformation of residual subgrains into grains, the partial finalization of recrystallization and gradual grain growth.

## 4. Discussion

The microstructure of the F0 material was not modified by the thermal treatment because this specimen was already annealed at high temperature that results in the formation of uniform recrystallized structure with stable phase composition. The significant increase of microhardness that takes place in the ARB processed materials is in accordance with LOM and TEM results due to the microstructure refinement occurring during ARB process. The F3 specimen contains very small (sub) grains of a submicron size (average length less than ~0.7 μm, average width ~0.5 μm) elongated in the rolling direction.

Huang *et al.* [20] have shown that in heavily deformed aluminium materials (also materials after the ARB process) the partial recovery of the dislocation substructure, which is coupled with the dislocation density decrease and changes in the low-angle subgrain boundary fraction, may result in a surprising increase of the strength of the material during annealing at elevated temperatures. Such a behavior was observed also in the material F3 (and F5) where the microhardness increases until a maximal value at about 160 °C was reached. In this interval of annealing temperatures the TEM observations show that the dislocation substructure is nearly fully recovered. Thus the emission of new dislocations necessary for the accommodation of deformation during subsequent indentation at room temperature is more difficult because only sparse cell walls and dislocations acting as new dislocation sources are present in the material. A (sub) grain growth that is observed by TEM above 200 °C is a concurrent process, which finally prevails above the strengthening described by Huang *et al.* [20], and the microhardness decreases again at higher annealing temperatures.

Fast drop of microhardness between 200 °C and 300 °C appears as a result of progressing recrystallization, collapse of the lamellar microstructure and spheroidisation of grains. The *in situ* experiments moreover show that the recrystallization does not proceed by a motion of the recrystallization front, but by a continuous (sub) grain growth. Typical example of such a transformation is the group of (sub) grains in the center of the Figure 9 marked by a circle. The outer boundary of this group resists the pressure of surrounding larger grains and finally (Figure 9d) one of the grain start to grow by the boundary migration and absorbs the neighboring grain. Such a phenomenon is known as continuous static recrystallization [21].

In comparison with direct-chill cast materials, no particle stimulated nucleation (PSN), which might positively randomize the final texture [22], but also can be a source of unfavorable abnormal grain growth, was observed. The absence of PSN is due to the homogeneous distribution of fine particles with the diameter less than 1 µm typical for TRC aluminium alloys. The 1 µm particle size is often mentioned as the lowest limit for the occurrence of PSN [23].

Above 350 °C the microhardness varies only modestly because the microstructure is almost fully recrystallized at 350 °C and only a recrystallization finalization and a moderate coarsening of grains occur at higher annealing temperatures. Nevertheless, the resulting microstructure still exhibits the presence of uniform fine grain size with the diameter not exceeding 5–10 µm, which makes the material a good candidate for further mechanical post-processing.

## 5. Conclusions

Thermal stability and static softening of a commercial TRC AA8006 type alloy after ARB treatment were studied. The significant increase of microhardness, which takes place in the ARB processed materials, is in accordance with LOM and TEM experiments due to the microstructure refinement occurring during ARB process. Local increase of microhardness appearing during first stages of annealing at temperatures below 160 °C is induced by the progressing recovery causing dislocation exhaustion and more difficult dislocation sources activation. Rearrangement of subgrain and grain boundaries followed by continuous recrystallization finally results in the degradation of the material microhardness and formation of uniform recrystallized microstructure.

## References

[1] Petch N.J. (1953). The cleavage strength of polycrystals. J. Iron Inst..

[2] Hall E.O. (1951). The deformation and ageing of mild steel. III Discussion of results. Oric. Phys. Soc..

[3] Valiev R.Z., Korznikov A.V., Mulyukov R.R. (1993). Structure and properties of ultrafine-grained materials produced by severe plastic deformation. Mater. Sci. Eng..

[4] Islamgaliev R.K., Chmelik F., Gibadullin I.F., Biegel W., Valiev R.Z. (1994). The nanocrystalline structure formation in germanium subjected to severe plastic deformation. Nanostruct. Mater..

[5] Saito Y., Utsunomiya H., Tsuji N., Sakai T. (1999). Novel ultra-high straining process for bulk materials—Development of the accumulative roll-bonding (ARB) process. Acta Mater..

[6] Kashihara K., Tsujimoto Y., Terada D., Tsuji N. (2013). Texture evolution in {112} <111> aluminum single crystals processed by severe plastic deformation. Mater. Charact..

[7] Schmidt C.W., Knödler P., Höppel H.W., Göken M. (2011). Particle based alloying by accumulative roll bonding in the system Al-Cu. Metals.

[8] Slámová M., Sláma P., Homola P., Uhlíř J., Cieslar M. (2009). Multilayer composite Al99.99/AlMg3 sheets prepared by accumulative roll bonding. Int. J. Mater. Res..

[9] Valiev R.Z., Islamgaliev R.K., Alexandrov I.V. (2000). Bulk nanostructured materials from severe plastic deformation. Prog. Mater. Sci..

[10] Valiev R.Z., Langdon T.G. (2006). Using high-pressure torsion for metal processing: Fundamentals and application. Prog. Mater. Sci..

[11] Huang X., Tsuji N., Hansen N., Minamino Y. (2003). Microstructural evolution during accumulative roll-bonding of commercial purity aluminum. Mater. Sci. Eng..

[12] Slámová M., Sláma P., Cieslar M. (2006). The influence of alloy composition on phase transformations and recrystallization in twin-roll cast Al-Mn-Fe alloys. Mater. Sci. Forum.

[13] Birol Y. (2008). Thermomechanical processing of a twin-roll cast Al–1Fe–0.2Si alloy. J. Mater. Process. Technol..

[14] Slámová M., Karlík M., Cieslar M., Chalupa B., Merle P. (2003). Structure transformations during annealing of twin-roll cast Al-Fe-Mn-Si (AA 8006) alloy sheets II. Effect of homogenization and heating rate. Kovove Mater..

[15] Cieslar M., Slámová M., Uhlíř J., Coupeau C.H., Bonneville J. (2007). Effect of composition and work hardening on solid solution decomposition in twin-roll cast Al-Mn sheets. Kov. Mater..

[16] Birol Y. (2009). Impact of homogenization on recrystallization of a supersaturated Al–Mn alloy. Scr. Mater..

[17] Homola P., Slámová M., Sláma P., Cieslar M. (2008). Thermal stability of ultrafine grains in Al-Fe-Mn-Si foils prepared by ARB and subsequent rolling. Mater. Sci. Forum.

[18] Karlík M., Slámová M., Homola P., Sláma P., Cieslar M. (2007). Accumulative roll-bonding (ARB) of sheets of aluminium and its commercial alloys AA8006 and AA5754 at ambient and elevated temperatures. Mater. Sci. Forum.

[19] Ham R.K. (1961). The determination of dislocation densities in thin films. Philos. Mag..

[20] Huang X., Hansen N., Tsuji N. (2006). Hardening by annealing and softening by deformation in nanostructured metals. Science.

[21] Jazaeri H., Humphreys F.J. (2004). The transition from discontinuous to continuous recrystallization in some aluminium alloys: II–Annealing behaviour. Acta Mater..

[22] Engler O., Vatne H.E., Nes E. (1996). The roles of oriented nucleation and oriented growth on recrystallization textures in commercial purity aluminium. Mater. Sci. Eng..

[23] Humphreys F.J., Hatherly M. (2004). Recrystallization and Related Annealing Phenomena.

